# Survival First: How Citizens Prioritize Competing Climate‐Health Risk Countermeasures Under Fiscal Constraints

**DOI:** 10.1111/risa.70315

**Published:** 2026-07-24

**Authors:** Katsuya Tanaka, Kazuhide Akaishi

**Affiliations:** ^1^ Faculty of Economics Shiga University Hikone City Japan; ^2^ National Research Institute for Earth Science and Disaster Resilience Tsukuba Japan; ^3^ Mark O. Hatfield School of Government Portland State University Portland Oregon USA; ^4^ Japan Meteorological Agency Tokyo Japan; ^5^ Organization for Research Promotion Shiga University Hikone City Japan

**Keywords:** best–worst scaling, energy affordability, sleep quality, urban heat adaptation, willingness to pay (WTP)

## Abstract

When governments must allocate limited resources among competing climate‐health countermeasures, citizen risk priorities determine political feasibility—yet whether those priorities translate into fiscal support remains poorly understood. This study develops a hybrid best–worst scaling and contingent valuation framework that integrates individual‐level priority parameters into an acceptance model, enabling direct testing of whether preference intensity predicts willingness to pay. Applied to a large‐sample survey of residents in Yokohama, Japan—a rapidly aging metropolis facing escalating heat emergencies—the analysis reveals three findings. First, citizens exhibit a survival‐first hierarchy, decisively prioritizing immediate protective measures over long‐term structural interventions, with consensus varying sharply across countermeasure types. Second, a systematic priority‐WTP disconnect emerges: The priority parameters for the most valued measures show no statistically detectable association with acceptance of the proposed payment, consistent with a possible entitlement interpretation in which life‐saving services are perceived as government obligations rather than goods warranting additional taxation, while a group of lower‐ranked supplementary measures is jointly associated with greater fiscal support. Third, chronic quality‐of‐life burdens—particularly sleep disruption—are more consistently associated with willingness to pay than are acute health symptoms, while private cooling costs are negatively associated with support for public adaptation, consistent with a crowding‐out mechanism that may pose a regressive barrier to equitable financing. These findings suggest that preference intensity and fiscal support may operate through partially distinct evaluative pathways, implying that standard valuation methods relying on willingness to pay alone can systematically misrepresent the structure of public risk priorities.

## 1 Introduction

### 1.1 Risk Prioritization Under Competing Hazard Countermeasures

Public risk management requires governments to determine how limited resources should be allocated among competing protective measures. Research in the psychometric tradition demonstrates that public risk judgments diverge systematically from expert assessments, shaped by perceived dread, controllability, and catastrophic potential rather than statistical probability alone (Wachinger et al. 2013; Botzen et al. 2015). These cognitive biases mean that hazards producing concentrated, visible destruction gain political priority more readily than diffuse, health‐mediated risks whose impacts are distributed across populations and time (Kasperson et al. 2022). When multiple hazard countermeasures compete for the same budget, this divergence creates a prioritization problem: The measures that technical analyses identify as most cost‐effective may not be those that citizens consider most urgent, and the resulting mismatch can undermine both policy implementation and public trust in governance.

Urban extreme heat exemplifies this prioritization problem with unusual clarity. Municipal governments confronting intensifying heatwaves must choose among countermeasures that operate on fundamentally different timescales and mechanisms. Immediate interventions—such as emergency medical response, public cooling shelters, and warning systems—reduce mortality and morbidity during ongoing heat events by providing acute physiological relief (Ebi et al. 2021; Hess et al. 2023). In contrast, structural measures—including urban greening, shading infrastructure, and reflective surface materials—gradually modify exposure by altering the urban surface energy balance, producing ambient cooling effects that accumulate over years or decades (Ziter et al. 2019; Mughal et al. 2020). Because both types of measures draw on the same constrained municipal budget, citizens and policymakers alike face a temporal allocation dilemma: how much priority should be given to certain, immediate protection versus uncertain, delayed risk reduction?

This allocation dilemma is particularly acute in rapidly aging societies. Older adults exhibit higher physiological sensitivity to thermal stress and greater prevalence of chronic disease, amplifying hospitalization risk during heat events (Ebi et al. 2021; Mora et al. 2017). Empirical evidence from multiple countries shows that ambulance dispatches and emergency admissions surge within short periods of elevated temperature (Anderson and Bell 2010; Gasparrini et al. 2015), placing intense operational pressure on public institutions that must repeatedly mobilize response capacity each summer. In Japan, where demographic aging is among the most advanced globally and summer temperatures have risen sharply in recent decades, municipal authorities must manage heat not only as a long‐term environmental challenge but as an episodic emergency requiring readiness and surge capacity. This dual temporal character of the hazard—chronic exposure combined with acute crises—makes resource allocation inherently contested, as investment in one temporal dimension necessarily reduces capacity in the other.

Despite extensive physical modeling of structural cooling interventions (Akbari and Kolokotsa 2016; Mughal et al. 2020), empirical evidence on how the public prioritizes these competing measures remains remarkably limited. Risk perception research has documented general attitudes toward heat hazards (Wachinger et al. 2013; Taylor et al. 2014), but has not examined how citizens rank specific countermeasures against one another when resources are explicitly constrained. The social amplification of risk framework (Kasperson et al. 1988) suggests that public attention to hazards is shaped by institutional responses and media signals, yet how this amplification process operates when multiple protective options compete for finite budgets has not been empirically tested. Without evidence on public risk prioritization, policymakers design heat adaptation portfolios based on technical efficacy alone, risking public resistance and implementation failure when citizens perceive that their most pressing needs are being neglected in favor of measures whose benefits are distant and uncertain (Adger et al. 2013; Shi et al. 2016).

The consequences of this evidence gap extend beyond individual policy choices. Climate adaptation planning increasingly recognizes that technical effectiveness is a necessary but insufficient condition for successful implementation; political feasibility, public legitimacy, and fiscal sustainability are equally critical (IPCC 2022; Hallegatte et al. 2016). Adaptation portfolios that fail to reflect societal risk priorities face chronic underfunding, political backlash, and ultimately abandonment—outcomes that technical assessment alone cannot predict or prevent. Understanding how citizens prioritize competing countermeasures therefore provides an essential behavioral foundation for designing adaptation strategies that are both effective and governable.

### 1.2 The Gap Between Stated Priorities and Fiscal Support

A second, largely unexamined dimension of the prioritization problem concerns the relationship between expressed preferences and actual willingness to finance preferred measures. In risk analysis, it is well established that stated concern about a hazard does not automatically translate into support for costly protective action (Botzen et al. 2015). The “risk perception paradox” describes situations where high perceived risk coexists with low preparedness or low willingness to pay (WTP) for risk reduction (Wachinger et al. 2013). This paradox has been documented primarily for natural hazards such as floods and earthquakes, where high risk awareness frequently coexists with minimal investment in protective measures, but whether the same disconnection operates in the context of climate adaptation—where multiple countermeasures with different temporal profiles compete for the same budget—has not been empirically examined.

The relationship between risk prioritization and fiscal support is complicated by the cognitive categorization of public goods. Fiscal psychology research suggests that citizens distinguish between services perceived as fundamental government obligations and those perceived as discretionary amenities (Kirchler 2007; Slemrod 2007). When a risk reduction measure falls into the “entitlement” category—as basic emergency medical services might—citizens may strongly prioritize it while simultaneously rejecting additional taxation to fund it, reasoning that the government should finance such services through existing revenue. This creates a fiscal paradox where the most popular measures are precisely the hardest to fund through new revenue instruments, because preference intensity reflects perceived governmental duty rather than willingness to bear personal cost.

Existing valuation studies in climate adaptation typically estimate WTP for individual measures independently (e.g., Tanaka et al. 2022), implicitly treating preferences as additive and assuming that high valuation maps straightforwardly onto funding support. This approach leaves fundamentally unclear whether high priority within a portfolio translates into financial support when the cost is borne personally and when the valued measure competes with alternatives for the same resources. If the most preferred countermeasure is also the one citizens consider an existing government obligation, preference intensity may not generate marginal WTP, producing a systematic disconnect that standard single‐measure valuation cannot detect.

Methodologically, addressing this gap requires a framework that jointly captures relative prioritization and monetary valuation within the same decision context. Standard contingent valuation (CV) estimates WTP for a predefined policy but does not reveal how that policy was prioritized among alternatives. Conversely, best–worst scaling (BWS) elicits relative importance rankings with high discrimination but provides no monetary metric. Integrating these two methods—using individual‐level BWS priority parameters as covariates in a CV acceptance model—enables direct testing of whether preference intensity predicts fiscal support, and identifies specific measures where this link breaks down. This hybrid stated‐preference design has not previously been applied to the prioritization of climate‐health risk countermeasures, despite its potential to reveal the cognitive mechanisms that mediate between risk concern and collective action.

The practical implications of this methodological gap are substantial. If policymakers assume that the most preferred measures will also attract the most funding, they may design financing strategies that fail politically. Conversely, if some measures generate disproportionate WTP relative to their priority ranking, alternative funding architectures may be feasible. Distinguishing between entitlement‐category and amenity‐category measures is therefore essential for designing adaptation financing that aligns with public cognitive structures rather than working against them.

### 1.3 Lived Experience as a Driver of Risk Valuation

A third research gap concerns the experiential pathways through which climate risk becomes personally salient and generates demand for collective protection. Dual‐process theories of risk cognition distinguish between analytical processing of statistical information and experiential processing based on affect and personal encounter (Slovic et al. 2004; Weber 2006). The analytical system evaluates risk through probability and consequence assessments, while the experiential system generates rapid affective responses based on past encounters with the hazard. Applied to heat risk, this distinction implies that chronic, embodied experiences of thermal stress—not merely knowledge of mortality statistics—may drive support for public intervention through affective channels that bypass deliberative cost‐benefit reasoning.

Epidemiological studies focus predominantly on acute outcomes such as heatstroke mortality and emergency hospitalizations (Gasparrini et al. 2015; Vicedo‐Cabrera et al. 2021), and this acute framing dominates both scientific communication and policy discourse. However, urban residents in warming cities experience heat primarily as cumulative quality‐of‐life degradation rather than episodic medical emergencies. Disrupted sleep during tropical nights, reduced capacity for outdoor activity, diminished work productivity, and increased energy expenditure for private cooling constitute the daily experiential reality of urban heat for the vast majority of residents who never experience clinical heatstroke (Obradovich et al. 2017). These chronic burdens may function as powerful experiential signals that translate abstract climate risk into concrete, personally felt economic demand for collective protection—yet their role in shaping adaptation WTP has received remarkably little empirical attention compared to acute risk indicators.

If chronic quality‐of‐life impacts indeed drive economic valuation more powerfully than acute health risk perceptions, current adaptation frameworks may systematically misallocate attention and resources. Policies designed around mortality reduction—while critically important—may fail to mobilize broad public support if the experiential drivers of that support are rooted in everyday thermal discomfort, sleep deprivation, and energy costs rather than in fear of death. Understanding which experiential pathway dominates WTP formation is therefore essential not only for accurate valuation but for effective risk communication and political strategy.

Furthermore, private cooling costs introduce a potential crowding‐out mechanism with important distributional implications. Households already burdened by rising electricity bills for air conditioning may oppose additional public spending on heat adaptation, even if they recognize the underlying risk (Davis and Gertler 2015; Barreca et al. 2016). Air conditioning functions as a private substitute for collective environmental protection, but its costs are not uniformly distributed: Lower‐income households in poorly insulated housing face disproportionate energy expenditure (Harlan et al. 2006; Santamouris et al. 2015). This interaction between private coping expenditure and support for collective risk reduction has been theorized in the adaptation literature but lacks empirical quantification at the household level. If energy affordability constrains fiscal support for public adaptation, and if electricity‐cost burdens are concentrated among lower‐income households living in poorly insulated housing, this relationship could create a regressive dynamic that undermines both equity and effectiveness.

### 1.4 Study Objective and Contribution

This study investigates how citizens prioritize competing urban heat countermeasures and whether those priorities translate into willingness to finance implementation. We examine this problem in Yokohama, Japan—a high‐density, rapidly aging metropolitan area of 3.77 million residents where summer heat increasingly generates emergency medical demand. The empirical analysis employs a hybrid stated‐preference survey combining BWS and CV, using individual‐level posterior means from the mixed‐logit BWS model as covariates in a binary logistic CV acceptance model.

The study contributes to risk analysis in three ways. First, it provides empirical evidence on how citizens prioritize competing risk countermeasures under explicit resource constraints, revealing a hierarchical preference structure that we term the “survival‐first” pattern. Unlike existing studies that assess attitudes toward heat in general terms, this analysis reconstructs the prioritization process itself, identifying which measures retain support when alternatives must be explicitly excluded. Second, it demonstrates that preference intensity does not uniformly predict fiscal support, identifying a systematic disconnect between prioritization and WTP for the most preferred measures—consistent with a possible entitlement interpretation in which life‐saving services are perceived as non‐negotiable government obligations rather than goods for which additional payment is appropriate. Third, it shows that chronic quality‐of‐life burdens—particularly sleep disruption—are more consistently associated with adaptation WTP than the measured acute health symptoms, connecting experiential risk processing theory to fiscal support for collective climate protection.

Together, these contributions address a question that is both theoretically important and practically urgent: Under what conditions does public concern about a risk translate into collective willingness to act, and what explains the persistent gap between the two?

## 2 Methods

### 2.1 Study Context

**FIGURE 1:**
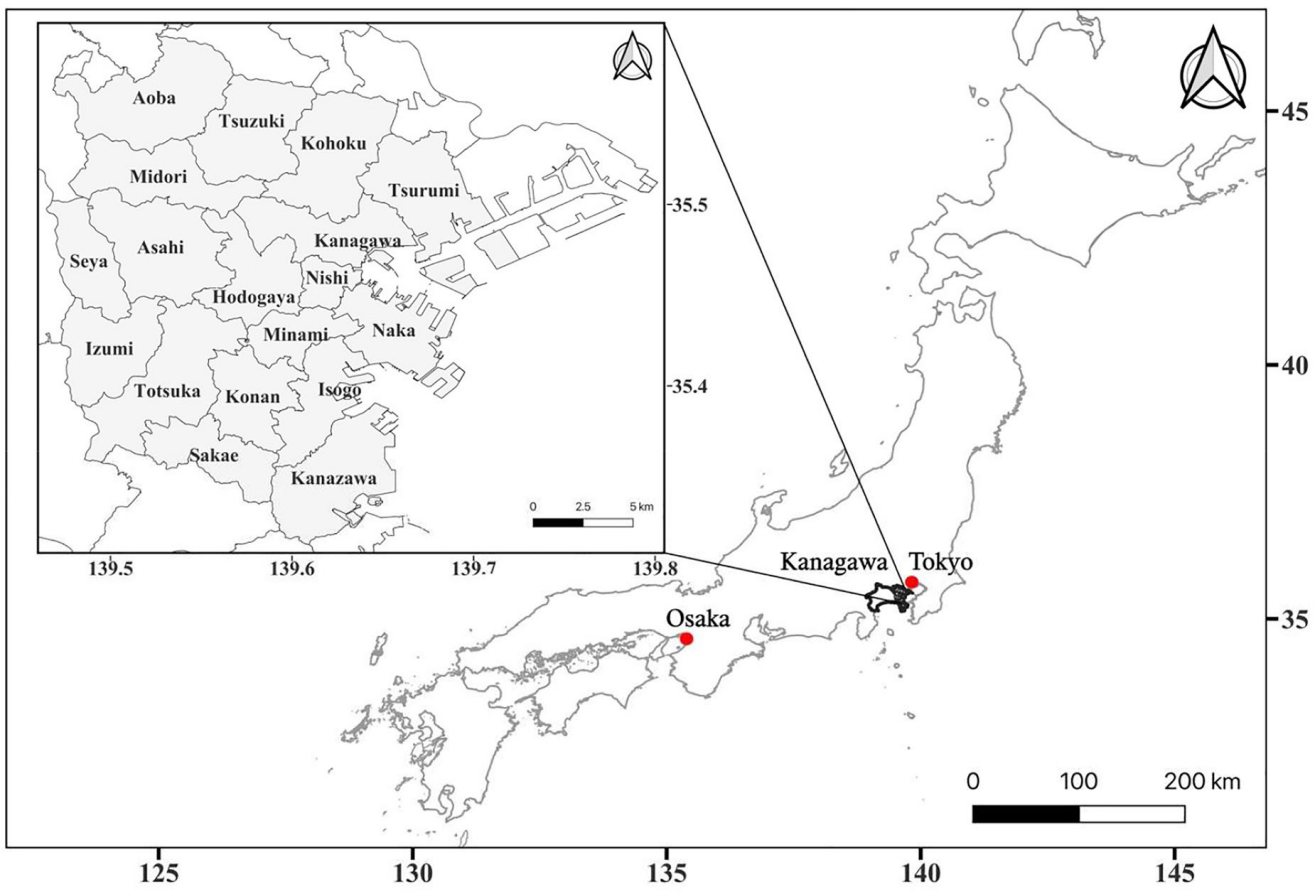
Study region (City of Yokohama, Kanagawa Prefecture).

This study examines the City of Yokohama (Figure 1), Japan's second‐largest municipality with approximately 3.77 million residents distributed across 18 administrative wards. Located within the Tokyo metropolitan region, Yokohama serves as a major economic center hosting manufacturing, logistics, and advanced service industries. The city's adaptation decisions therefore carry consequences extending beyond municipal boundaries, as disruptions in daily urban functioning propagate through regional economic networks.

Yokohama faces intensifying heat stress from the combined effects of climate change and the Urban Heat Island (UHI) phenomenon. Meteorological observations indicate a persistent rise in summer temperatures, with days exceeding 35°C becoming increasingly frequent. The number of tropical nights—during which nighttime minimum temperatures remain above 25°C—rose from approximately 18 days annually (1981–2010 average) to nearly 30 days (1991–2020 average), and exceeded 60 days annually for three consecutive years from 2023 to 2025 (Japan Meteorological Agency 2025). This persistent nocturnal heat prevents physiological recovery and amplifies cumulative exposure in dense residential districts, creating health risks that extend well beyond the daytime hours typically emphasized in heat warnings.

The public health implications of these climatic trends are already evident. According to administrative records published by the City of Yokohama, heatstroke‐related emergency medical transportations during the summer months (May through September) rose sharply from 661 cases in 2016 to 1,727 cases in 2025—an increase of more than 2.5 times within less than a decade (City of Yokohama n.d.). Extreme heat has thus transitioned from an occasional environmental hazard to a recurrent operational burden on emergency services, requiring repeated mobilization of response capacity while simultaneously demanding consideration of preventive measures capable of reducing future demand.

This coexistence of recurrent emergency demand and escalating long‐term exposure creates a concrete allocation problem. Municipal governments operating under fiscal constraints cannot expand all countermeasures simultaneously. Investments in emergency medical capacity, cooling shelters, and warning systems compete directly with structural interventions such as urban greening and shading infrastructure. Yokohama therefore provides an empirical setting in which the trade‐off between immediate response and preventive adaptation is not hypothetical but institutionally unavoidable. Although the analysis focuses on a single city, the conditions observed in Yokohama—aging populations, concentrated infrastructure dependence, and warming climates—are increasingly common among large metropolitan areas worldwide, making the findings relevant to a broader class of urban adaptation challenges.

### 2.2 Survey Design

**FIGURE 2:**
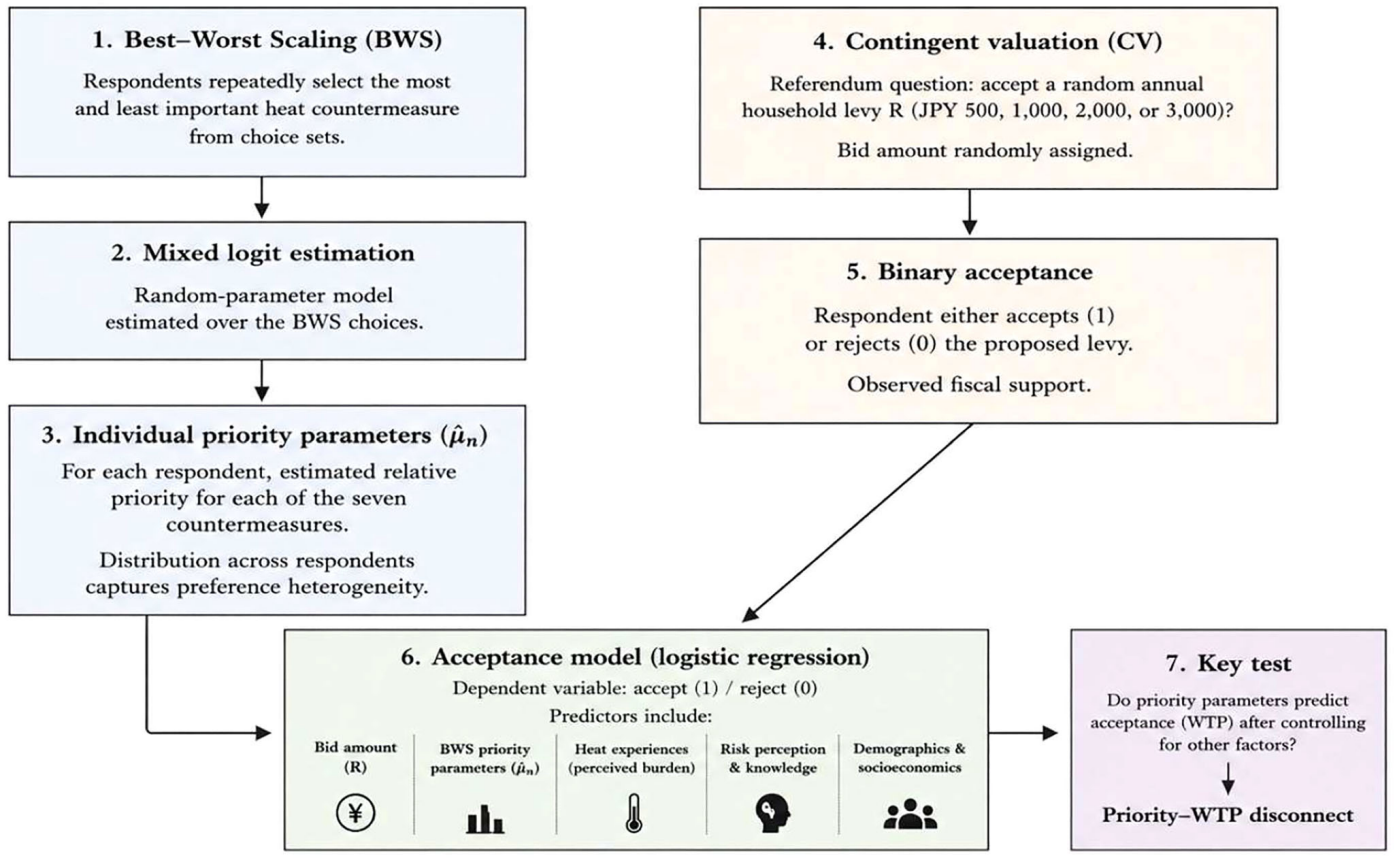
Schematic of the hybrid Best‐Worst Scaling and contingent valuation (BWS–CV) framework. *Note*: The framework links revealed priorities from BWS to stated fiscal support from CV to examine whether the countermeasures people prioritize most are also those they are willing to pay for.

The survey instrument was designed to approximate a collective decision environment in which multiple heat adaptation measures compete for limited public resources. Rather than evaluating single interventions independently, respondents were asked to consider a portfolio of potential policies under a shared municipal framework. The questionnaire therefore functions not only as a valuation tool but as a behavioral observation device capturing prioritization under constraint. Figure 2 summarizes the hybrid best–worst scaling and contingent valuation (BWS–CV) design used to elicit and test these priorities.

The first section collected information on heat exposure and lived experience during the previous summer. Respondents reported health symptoms (including heatstroke‐like symptoms and sleep disturbance), daily life disruptions (such as difficulty performing outdoor activities and declining work performance), and economic burdens (particularly increased electricity costs for cooling). These variables represent the experiential pathways through which climate risk becomes personally salient and provide behavioral context for interpreting subsequent policy choices. The section also assessed familiarity with official heat warnings and knowledge of temperature indices to capture differences in informational exposure.

The second section introduced eight public countermeasures: (1) *Strengthening emergency medical services*, (2) *expanding public cooling shelters*, (3) disseminating real‐time heat information, (4) *promoting urban greening*, (5) *creating shaded areas*, (6) *installing public water refill stations*, (7) *implementing public education on heat prevention*, and (8) *refining heat alert systems* (Table 1). Each measure was described concisely to ensure comparable interpretation across respondents. Instead of rating items independently, participants repeatedly selected the most and least important options from subsets of policies using BWS (Section 2.3). This structure forces respondents to make trade‐offs and thereby reveals relative prioritization when not all measures can be equally supported.

**TABLE 1:** Description of public heat countermeasures used in the survey.

Measure	Description	Category
Strengthening emergency medical services	Enhancing coordination with medical institutions and emergency services. This aims to establish a system for the early detection of heatstroke patients and rapid, effective medical response.	Immediate protective
Expanding public cooling shelters	Supporting the creation of designated cooling spaces in shopping districts and malls. These shelters allow citizens to cool down and rest during daily activities such as shopping or walking.	Immediate protective
Disseminating clear real‐time heat information	Distributing heatstroke information and alerts via smartphone apps and emails in real time. This ensures accessibility for all citizens, helping them obtain necessary information in advance to take preventive measures.	Immediate (information)
Promoting urban greening	Increasing street trees and greening building rooftops to mitigate urban heat islands and cool the entire city. These green areas serve as recreational spaces for citizens and provide natural cooling effects.	Long‐term structural
Creating shaded areas	Establishing shaded spaces in parks and along sidewalks to provide temporary refuge from intense sunlight. These spots offer a safe resting environment for citizens during hot weather.	Long‐term structural
Installing public water refill stations	Setting up free water dispensers in public areas. This infrastructure facilitates immediate hydration when citizens feel hot, thereby reducing the risk of heatstroke while outdoors.	Immediate (supplementary)
Implementing public education on heat prevention	Conducting educational programs in schools, workplaces, and communities to disseminate correct knowledge and prevention methods. The goal is to build a community where every citizen understands how to prevent and respond to heatstroke.	Preventive (information)
Refining heat alert systems	Enhancing the precision and timeliness of announcements regarding high‐risk locations and times. Alerts are designed to be specific and easily understood to enable prompt action by citizens.	Immediate (information)

The third section introduced a collective financing scenario. Respondents were asked whether they would support implementation of the measures they had prioritized if funded through an annual household tax increase (Section 2.4). The payment amount was randomly varied across participants. This step links prioritization to fiscal acceptance, allowing observation of whether preferred measures remain supported when personal cost is introduced.

Prior to deployment, the questionnaire was pilot‐tested to ensure comprehension and stable response patterns. The final survey was administered online to enable interactive choice tasks while maintaining consistent visual presentation. Participation was voluntary and anonymous, and informed consent was obtained from all respondents.

### 2.3 BWS: Eliciting Risk Prioritization

To identify how residents prioritize competing heat countermeasures, we employed the BWS Case 1 approach (Louviere et al. 2015). The objective of BWS in this context is not simply to rank amenities but to reconstruct a prioritization process in which not all policies can be simultaneously supported. Unlike Likert‐type ratings that allow respondents to endorse every safety measure equally, BWS forces exclusion: Participants must identify which measures they consider most and least important within each choice set. The method therefore reveals which measures retain support when attention and resources must be allocated selectively, approximating the constrained decision environment that characterizes actual policy choice.

**FIGURE 3:**
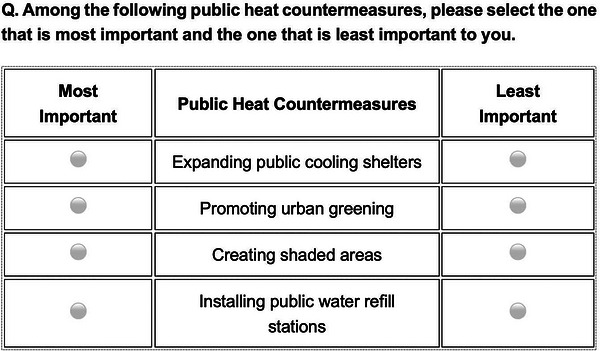
Example of a Best‐Worst Scaling (BWS) choice set.

Respondents evaluated repeated choice sets, each containing four countermeasures randomly drawn from the full list of eight alternatives. Within each set, they identified the most important and least important measure (Figure 3). The combinations followed a balanced incomplete block design to ensure that each measure appeared with equal frequency and in comparable contexts across respondents. Each respondent completed four choice sets, generating a total of 7,276 choice occasions across the sample. The repeated forced‐choice structure approximates a constrained policy discussion in which alternatives compete for priority rather than receiving approval in isolation.

The BWS choice data were analyzed within the Random Utility Maximization (RUM) framework (McFadden 1973). Let $U_{njt}$ denote the utility derived by individual *n* from countermeasure *j* in choice task *t*. The probability that respondent *n* selects alternative *i* as best and alternative *k* as worst from a given set is specified by the conditional logit (CL) model:

(1) $$P_{nt}\;\left(i,k\right)=\frac{\exp\left(\mu_{i}\;-\;\mu_{k}\right)}{\sum_{p=1}^{J}\sum_{\begin{array}{c} q=1\\ q\;\neq \;p \end{array}}^{J}\exp\left(\mu_{p}\;-\;\mu_{q}\right)}\;$$

where $\mu_{j}$ represents the relative priority assigned to measure *j*. Rather than representing absolute utility, these parameters describe comparative support once alternatives compete for limited attention.

To capture heterogeneity in prioritization across residents, the model was extended to a mixed logit (ML) specification (Train 2009). Individual priority parameters $\mu_{n}$ follow a distribution f(μ|θ), yielding conditional probability

(2) $$P_{n}\;\left(i,k|\mu_{n}\right)=\frac{\exp\left(\mu_{\mathrm{ni}}\;-\;\mu_{nk}\right)}{\sum_{p=1}^{J}\sum_{\begin{array}{c} q=1\\ q\;\neq \;p \end{array}}^{J}\exp\left(\mu_{np\;}-\;\mu_{nq}\right)}\;$$

and unconditional likelihood

(3) $$L_{n}\;\left(\theta \right)=\int \prod_{t=1}^{T}P_{n}\left(i_{t},k_{t}|\mu_{n}\right)f\left(\mu_{n}|\theta \right)\;d\mu_{n}$$

The estimated individual mean parameters ${\hat{\mu }}_{n}$ represent each respondent's prioritization structure and are subsequently used to examine whether preferred measures remain supported once fiscal cost is introduced (Section 2.4).

### 2.4 CV: Testing Whether Priorities Survive Cost

The CV module estimates WTP for the implementation of the respondent‐prioritized policy package. In this framework, WTP is interpreted as an indicator of fiscal support for collective risk reduction rather than as the isolated economic value of individual environmental amenities. The key analytical innovation is the integration of BWS‐derived priority parameters into the CV acceptance model, which enables direct testing of whether measures that citizens rank as most important are also the ones they are willing to finance through personal taxation.

**FIGURE 4:**
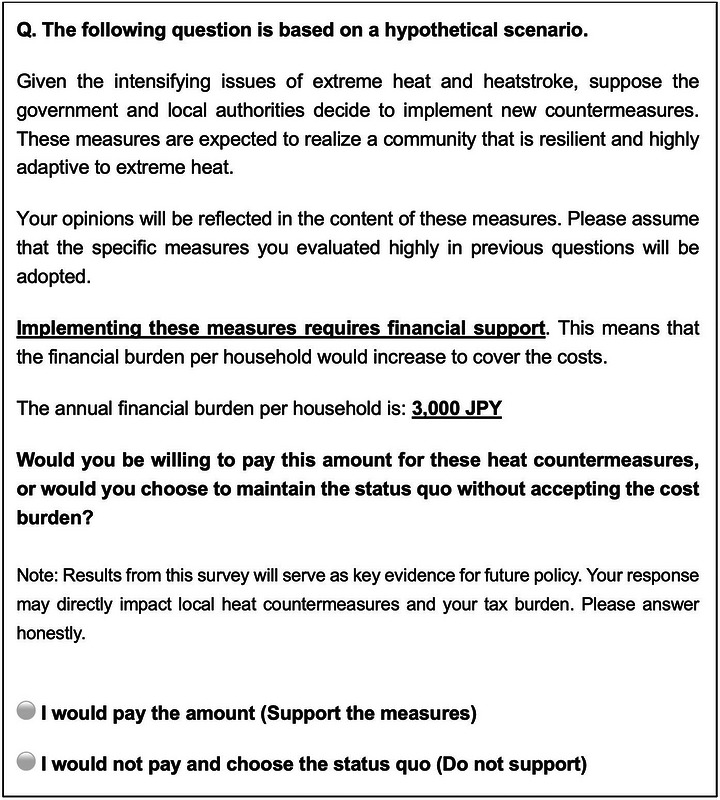
Example of the hypothetical scenario and willingness‐to‐pay (WTP) question.

Respondents were presented with a policy scenario titled Comprehensive Heat Adaptation Plan (Figure 4). The scenario explicitly stated that the municipality would implement the countermeasures the respondent had prioritized in the BWS section, thereby linking the valuation target to each respondent's own preference structure. Each respondent received a randomly assigned bid amount R of JPY 500, 1,000, 2,000, or 3,000^1^ and answered a binary referendum‐style question asking whether they would support the policy at that cost. This single‐bounded dichotomous choice format follows established CV practice (Arrow et al. 1993) and allows estimation of both acceptance probability and WTP under a format designed to reduce strategic response incentives.

The decision is modeled as a comparison between utility with and without policy implementation. The probability that respondent *i* supports the policy is specified as:

(4) $$P_{i}=\frac{1}{1\;+\exp\left(-\left(X_{i}^{\prime }\beta \;-\;\gamma R\right)\right)}\;$$

where $X_{i}^{\prime }\beta$ represents the perceived benefit of the adaptation package and includes experiential variables (sleep disturbance, electricity burden, outdoor activity limitations), risk perception measures, demographic characteristics, and critically, the individual‐level BWS priority parameters ${\hat{\mu }}_{n}$ estimated from the ML model. The term γR captures the disutility of payment, where γ>0 denotes the marginal utility of income.

The integration of BWS parameters into the CV model constitutes the key methodological contribution of this study. If preference intensity uniformly predicted WTP, all BWS parameters would enter the acceptance model with positive and significant coefficients—indicating that respondents who more strongly prioritize a given measure are also more willing to pay for the overall adaptation package. We assess this pattern using the individual coefficients, joint Wald tests for the three highest‐ and the three lower‐ranked measures, and a linear contrast comparing the mean coefficients of the two groups. A disconnect is inferred when the highly ranked measures show no detectable joint association with acceptance while the lower‐ranked measures show a significantly stronger positive association. Such disconnects may indicate entitlement perceptions (where citizens view a service as a government obligation that should not require new taxation), payment vehicle resistance (where new levies are rejected regardless of the valued outcome), or substitution effects (where private coping reduces demand for collective provision). The hybrid framework thus moves beyond the standard CV question of “how much will you pay?” to ask “does your ranking predict what you will pay?”—a question that single‐method designs cannot address.

Individual implied WTP is computed following Tanaka et al. (2022):

(5) $$E\;\left(\mathrm{WT}P_{i}\right)=-\frac{1}{\gamma }ln\left(1\;+\exp\left(-X_{i}^{\prime }\beta \right)\right)$$

All variables included in the estimation, together with their definitions and coding, are summarized in Table 2. Because household income had substantial non‐response (22.2% declined to answer), it enters the model as indicators for low (below JPY 4 million), high (JPY 8 million or more), and unreported income, with middle income (JPY 4–8 million) as the reference category, so that all 1,819 observations are retained without imputation. The low‐ and high‐income indicators are not jointly significant in the acceptance model; the full income specification is reported in the Supporting Information (Table S4).

**TABLE 2:** Definition and descriptive statistics of predictor variables.

Category	Variable	Mean	SD	Min.	Max.
**Dependent variable**					
Acceptance	Accepting the proposed payment (binary)	0.57	—	0	1
**Independent variables**					
Cost	Annual payment amount (JPY)	1,584.4	964.4	500	3,000
Experiences during heatwaves in 2023	Difficulty performing outdoor activities	0.43	—	0	1
	Decline in work or academic performance	0.25	—	0	1
	Experienced heatstroke‐like symptoms outdoors	0.14	—	0	1
	Experienced heatstroke‐like symptoms indoors	0.09	—	0	1
	Experienced sunburn or skin problems	0.13	—	0	1
	Reduced sleep quality (e.g., difficulty sleeping)	0.35	—	0	1
	Loss of appetite or weight change	0.10	—	0	1
	Burden of increased electricity costs	0.61	—	0	1
Preferences for heat countermeasures	Strengthening emergency medical services	2.71	1.47	−2.29	7.99
	Expanding public cooling shelters	1.82	2.05	−6.98	8.73
	Disseminating clear real‐time heat information	1.29	1.32	−3.32	6.76
	Promoting urban greening	1.22	2.50	−6.97	10.21
	Creating shaded areas	0.76	1.59	−5.83	6.15
	Installing public water refill stations	0.46	2.10	−5.58	7.21
	Implementing public education on heat prevention	0.38	1.40	−4.93	4.96
Heat perception and knowledge	Perception: Unprecedented extreme heat is likely to occur	0.44	—	0	1
	Knowledge: Familiarity with Heatstroke Alert (1–4 scale)	2.72	0.73	1	4
	Knowledge: Unaware of WBGT (lowest familiarity)	0.25	—	0	1
	Knowledge: Highly familiar with WBGT (highest familiarity)	0.06	—	0	1
Demographics and socioeconomic	Gender: Male	0.55	—	0	1
	Age: Young adults (18–34 years old)	0.15	—	0	1
	Age: Elderly (65 years or older)	0.21	—	0	1
	Housing: Resides in a single‐family home	0.34	—	0	1
	Risk tolerance (0–10 scale; higher = risk seeking)	5.07	2.25	0	10
	Income: Low (below JPY 4 million)	0.29	—	0	1
	Income: High (JPY 8 million or more)	0.16	—	0	1
	Income: Not reported	0.22	—	0	1
	Education (1–7 scale; higher = more education)	4.14	1.03	2	7

*Note*: All variables except the annual payment amount, the preference parameters, risk tolerance, Heatstroke‐Alert familiarity (1–4) and education (1–7) are coded as 0 = ‘No’ and 1 = ‘Yes.’ Variables under “Preferences for Heat Countermeasures” represent the individual‐specific posterior means derived from the mixed logit model estimation.

### 2.5 Survey Overview and Sample Demographics

The survey was conducted in March 2024, targeting residents aged 18 years or older living in Yokohama City, Japan. Respondents were recruited through a certified online research panel administered by Intage Inc., one of the largest survey firms in Japan. The online format enabled the implementation of interactive BWS choice tasks and CV scenarios while maintaining consistent visual presentation across respondents. To approximate population representativeness, stratified sampling was employed based on gender and age categories following the 2020 national census distribution.

A total of 1,819 valid responses were obtained after excluding incomplete or inconsistent answers. Table 3 compares the demographic composition of the final sample with official statistics for the adult population aged 18 and older (*N* = 3,163,409; the city's total resident population is approximately 3.77 million). Overall, the sample broadly reflects the demographic structure of the city. Gender distribution is reasonably balanced (sample: 56.2% male; population: 49.2%), and all major age groups are represented. Older residents—the group most physiologically vulnerable to heat‐related illness—account for 21.1% of the sample, allowing adaptation preferences among this high‐risk group to be examined.

**TABLE 3:** Comparison between sample and population characteristics.

	Population (*N* = 3,163,409)		Sample (*n* = 1,819)	
Variable	Frequency	%	Frequency	%
**Gender**				
Male	1,555,258	49.2	1,008	56.2
Female	1,608,151	50.8	787	43.8
**Age**				
18–24	205,395	6.5	61	3.4
25–34	428,381	13.5	213	11.9
35–44	464,166	14.7	368	20.5
45–54	610,083	19.3	440	24.5
55–64	516,361	16.3	334	18.6
65+	939,023	29.7	378	21.1
**Education**				
College degree or higher	1,260,000	39.8	997	54.8
**Annual household income**				
Less than JPY 2 million	—	—	183	10.1
JPY 2–4 million	—	—	347	19.1
JPY 4–6 million	—	—	368	20.2
JPY 6–8 million	—	—	224	12.3
JPY 8–10 million	—	—	144	7.9
JPY 10–15 million	—	—	102	5.6
JPY 15–20 million	—	—	32	1.8
JPY 20 million or more	—	—	16	0.9
Declined to answer	—	—	403	22.2

*Note*: Population figures are derived from the 2020 Population Census (Statistics Bureau of Japan 2021) and represent the adult population (aged 18 and older). Individuals with unknown age are excluded. Comparable population income statistics are not available from this census tabulation, so the income distribution is reported for the sample only. Gender and age categories may not sum to 1,819 because some respondents preferred not to state their exact gender or age.

Several systematic deviations typical of online panels are nonetheless observed. Younger residents (18–24) are underrepresented, while middle‐aged cohorts (35–54) are relatively overrepresented. More notably, educational attainment is substantially higher in the sample (54.8% college graduates) than in the population (39.8%). Because education and socioeconomic status are positively associated with environmental risk awareness and WTP, this composition likely introduces an upward bias in estimated absolute WTP levels. Furthermore, the online format inherently excludes digitally disconnected populations, who may be disproportionately elderly, low‐income, and vulnerable to both heat exposure and energy poverty.

Within‐respondent comparisons may reduce some response‐style biases, but they do not eliminate possible selection bias in the aggregate preference ordering. The BWS component relies on within‐respondent comparisons and is therefore robust to uniform socioeconomic scaling effects. Likewise, the CV model estimates marginal effects conditional on individual characteristics, allowing interpretation in behavioral rather than purely monetary terms. Consequently, while absolute WTP magnitudes should be interpreted conservatively, the inferred prioritization structure and the determinants of fiscal support—which are the primary analytical objectives—should be generalized only cautiously to other settings.

## 3 Results

### 3.1 Risk Prioritization: The Survival‐First Hierarchy

Table 4 presents the aggregate prioritization of heat countermeasures using the BWS counting analysis. The best–worst score for each countermeasure is defined as the frequency with which an option was selected as the most important (best) minus the frequency with which it was selected as the least important (worst). The table also reports the best/worst ratio as a complementary indicator of the balance between positive and negative selections.

**TABLE 4:** Results of Best‐Worst Scaling count analysis for public heat countermeasures.

Public heat countermeasures	Best	Worst	B–W	B/W
Strengthening emergency medical services	1,573	206	1,367	7.6
Expanding public cooling shelters	1,125	772	353	1.5
Disseminating clear real‐time heat information	978	823	155	1.2
Promoting urban greening	961	1,116	−155	0.9
Creating shaded areas	901	941	−40	1.0
Installing public water refill stations	757	1,229	−472	0.6
Implementing public education on heat prevention	598	1,019	−421	0.6
Refining heat alert systems	383	1,170	−787	0.3

The BWS count results show a clear separation between the highest‐ranked and lowest‐ranked measures. *Strengthening emergency medical services* records the highest best–worst score (1,367) and the highest B/W ratio (7.6), indicating that this measure was selected as “Best” nearly eight times more frequently than as “Worst.” *Expanding public cooling shelters* ranks second (B‐W = 353; B/W = 1.5), followed by *Disseminating clear real‐time heat information* (B‐W = 155; B/W = 1.2). In contrast, *Refining heat alert systems* records the lowest score (B‐W = −787; B/W = 0.3), indicating that it was selected as “Worst” far more frequently than as “Best.” The remaining measures form a middle‐to‐lower tier: *Creating shaded areas* is near zero (B‐W = −40; B/W = 1.0), *Promoting urban greening* is mildly negative (B‐W = −155; B/W = 0.9), while public education (B‐W = −421) and water refill stations (B‐W = −472) record more substantially negative values.

These rankings reveal a substantively meaningful three‐tier structure. The top tier comprises measures that provide direct, immediate physiological protection during heat events—emergency medical response and cooling shelters—and these are the only measures with strongly positive B‐W scores. The middle tier comprises an information measure (real‐time heat information) together with structural environmental measures (shaded areas and urban greening), whose B‐W scores cluster near zero, suggesting that the population is broadly balanced in their best and worst selections. The bottom tier contains measures perceived as either redundant with existing infrastructure (refined alert systems) or insufficient in scale relative to the threat (water refill stations, public education). This three‐tier pattern suggests that citizens evaluate heat countermeasures primarily through the lens of direct, high‐intensity protection against the serious health consequences of heat—rather than mere immediacy of availability, since some immediately usable measures such as water‐refill stations rank low—and the certainty of that protection, rather than through cost‐effectiveness or long‐term environmental benefit.

To account for choice trade‐offs within each task and to quantify preference heterogeneity across individuals, we estimated random utility models. Table 5 reports results from both a CL model and a ML model. Because the ML specification allows coefficients to vary across individuals through random parameter distributions, it captures unobserved heterogeneity not accommodated by the CL model. Model fit statistics confirm that the ML model fits the data substantially better than the CL model (Akaike Information Criterion, AIC: 33,100 vs. 38,147), and we therefore use the ML results as the primary basis for interpretation.

**TABLE 5:** Best‐Worst Scaling (BWS) estimation results for public heat countermeasures: Conditional and mixed logit models.

	Public heat countermeasures	Conditional logit (CL)	Mixed logit (ML)
**Mean parameters**	Strengthening emergency medical services	1.57^***^ (0.04)	2.66^***^ (0.08)
	Expanding public cooling shelters	1.06^***^ (0.04)	1.81^***^ (0.08)
	Disseminating clear real‐time heat information	0.80^***^ (0.04)	1.28^***^ (0.06)
	Promoting urban greening	0.77^***^ (0.04)	1.19^***^ (0.09)
	Creating shaded areas	0.50^***^ (0.04)	0.78^***^ (0.07)
	Installing public water refill stations	0.33^***^ (0.04)	0.46^***^ (0.08)
	Implementing public education on heat prevention	0.21^***^ (0.03)	0.39^***^ (0.06)
**S.D. parameters**	Strengthening emergency medical services		1.93^***^ (0.08)
	Expanding public cooling shelters		2.53^***^ (0.09)
	Disseminating clear real‐time heat information		1.75^***^ (0.07)
	Promoting urban greening		3.10^***^ (0.10)
	Creating shaded areas		2.12^***^ (0.08)
	Installing public water refill stations		2.58^***^ (0.08)
	Implementing public education on heat prevention		1.84^***^ (0.07)
	Number of observations	58,208	58,208
	Number of cases	7,276	7,276
	Number of respondents	1,819	1,819
	Log‐likelihood	−19,066	−16,536
	Akaike Information Criterion (AIC)	38,147	33,100

*Note*: Standard errors are in parentheses. The reference category is “Refining heat alert systems.”

*** *p* < 0.01.

With *Refining heat alert systems* as the reference category (coefficient normalized to zero), all other ML mean parameters are positive and statistically significant (*p* < 0.01). The largest mean coefficient is observed for *Strengthening emergency medical services* (*β* = 2.66), followed by *Expanding public cooling shelters* (*β* = 1.81) and *Disseminating clear real‐time heat information* (*β* = 1.28). *Promoting urban greening* (*β* = 1.19) and *Creating shaded areas* (*β* = 0.78) also show positive mean coefficients, while *Installing public water refill stations* (*β* = 0.46) and *Implementing public education on heat prevention* (*β* = 0.39) have smaller though still statistically significant positive magnitudes. This ordering is broadly consistent with the BWS counting results while providing magnitude‐based comparisons that incorporate within‐task trade‐offs.

The standard deviation (SD) parameters from the ML model reveal substantial and substantively important heterogeneity across individuals. All SD estimates are statistically significant (*p* < 0.01). *Promoting urban greening* exhibits the largest SD (*σ* = 3.10) relative to its mean (*β* = 1.19), yielding a coefficient of variation (*σ*/*β*) of 2.6—indicating comparatively high preference heterogeneity for this measure across the population. Relatively large SD estimates are also observed for *Installing public water refill stations* (*σ* = 2.58) and *Expanding public cooling shelters* (*σ* = 2.53). By contrast, *Strengthening emergency medical services* combines the highest mean (*β* = 2.66) with a comparatively smaller SD (*σ* = 1.93), yielding a coefficient of variation of 0.7. This pattern—high mean with moderate dispersion for immediate protective measures versus moderate mean with high dispersion for structural environmental measures—characterizes the “survival‐first” hierarchy and has direct implications for the political feasibility of different adaptation strategies.

### 3.2 Fiscal Support and WTP

Table 6 presents the logistic regression results for the CV acceptance model, in which the dependent variable is acceptance of the proposed annual household tax increase (1 = accept; 0 = reject). The model includes 27 covariates spanning cost sensitivity, heat‐related experiences, BWS‐derived preference parameters, risk perception and knowledge variables, and demographic and socioeconomic characteristics. We report fit relative to the naive baseline that classifies every respondent into the modal (accept) category, which is correct 57.1% of the time. The model attains McFadden's pseudo‐*R* ^2^ of 0.082 (Cox–Snell 0.106; Cragg–Uhler 0.142), an area under the ROC curve (c‐statistic) of 0.69, and 65.6% correct classification (log‐likelihood: −1,140.7; AIC: 2,341.4). The model's explanatory and predictive performance is modest. Accordingly, we interpret the model primarily as an explanatory model of associations rather than as a tool for predicting individual acceptance decisions. Because the model includes many predictors, we also verified that they are neither collinear nor reducible to coherent composite scales suitable for the model: All variance inflation factors are below 1.5 (mean ≈ 1.1), and exploratory factor analyses of the eight heat‐experience items and of the seven countermeasure‐preference parameters indicate that they cannot be collapsed into clean composite scales—the experience items load weakly on a single common factor with high item‐specific variance and include components with opposing effects on acceptance, whereas the preference parameters are multidimensional (three factors with eigenvalues above one). We therefore retain the individual variables; full diagnostics (variance inflation factors, correlation matrices, and scree plots) are reported in the Supporting Information (Tables S1–S3 and Figures S1 and S2).

**TABLE 6:** Logistic regression results for the acceptance of the proposed payment for public heat countermeasures.

Category	Variable	Coefficient	Odds ratio
Payment	Annual payment amount	−2.3E‐04^***^ (5.2E‐05)	1.000^***^ (5.2E‐05)
Experiences during heatwaves	Difficulty performing outdoor activities	0.246^**^ (0.105)	1.279^**^ (0.135)
in 2023	Decline in work or academic performance	−0.002 (0.123)	0.998 (0.123)
	Experienced heatstroke‐like symptoms outdoors	−0.278 (0.158)	0.757 (0.120)
	Experienced heatstroke‐like symptoms indoors	0.044 (0.185)	1.045 (0.193)
	Experienced sunburn or skin problems	0.164 (0.158)	1.178 (0.186)
	Reduced sleep quality (e.g., difficulty sleeping)	0.379^***^ (0.111)	1.461^***^ (0.162)
	Loss of appetite or weight change	0.092 (0.177)	1.097 (0.194)
	Burden of increased electricity costs	−0.319^***^ (0.105)	0.727^***^ (0.076)
Preferences for	Strengthening emergency medical services	−0.004 (0.034)	0.996 (0.034)
Public heat countermeasures	Expanding public cooling shelters	0.018 (0.026)	1.018 (0.026)
	Disseminating clear real‐time heat information	−0.005 (0.040)	0.995 (0.040)
	Promoting urban greening	0.040 (0.021)	1.041 (0.022)
	Creating shaded areas	−0.037 (0.033)	0.964 (0.032)
	Installing public water refill stations	0.064^**^ (0.025)	1.066^**^ (0.027)
	Implementing public education on heat prevention	0.115^***^ (0.037)	1.121^***^ (0.041)
Heat perception and knowledge	Perception: Unprecedented extreme heat is likely to occur	0.277^***^ (0.105)	1.319^***^ (0.139)
	Knowledge: Familiarity with Heatstroke Alert (1–4 scale)	0.337^***^ (0.080)	1.400^***^ (0.112)
	Knowledge: Unaware of WBGT (lowest familiarity)	−0.285^**^ (0.125)	0.752^**^ (0.094)
	Knowledge: Highly familiar with WBGT (highest familiarity)	−0.526^**^ (0.236)	0.591^**^ (0.140)
Demographics and socioeconomic	Gender: Male	0.069 (0.109)	1.071 (0.116)
	Age: Young adults (18–34 years old)	−0.302^**^ (0.148)	0.739^**^ (0.109)
	Age: Elderly (65 years or older)	0.595^***^ (0.141)	1.813^***^ (0.256)
	Housing: Resides in a single‐family home	0.280^**^ (0.110)	1.323^**^ (0.145)
	Risk tolerance (0–10 scale; higher = risk seeking)	0.092^***^ (0.023)	1.097^***^ (0.025)
	Income: Low (below JPY 4 million)	−0.099 (0.134)	0.906 (0.121)
	Income: High (JPY 8 million or more)	0.066 (0.156)	1.069 (0.167)
	Income: Not reported	−0.352^**^ (0.141)	0.703^**^ (0.099)
	Education (1–7 scale; higher = more education)	−0.117^**^ (0.050)	0.890^**^ (0.044)
	Constant	−0.502 (0.391)	
	Number of observations	1,819	
	Log‐likelihood	−1,140.7	
	AIC	2,341.4	
	McFadden's pseudo‐*R* ^2^	0.082	
	Cox–Snell/Cragg–Uhler (Nagelkerke) *R* ^2^	0.106/0.142	
	Area under ROC curve (c‐statistic)	0.690	
	Correct classification (baseline = 57.1%)	65.6%	

*Note*: Standard errors in parentheses. **p < 0.05, ***p < 0.01; significance is reported at the 5% and 1% levels only.

Abbreviation: WBGT = Wet bulb globe temperature.

As expected, the annual payment amount is negatively associated with acceptance and is statistically significant (*β* = −2.3E‐04, *p* < 0.01), confirming that respondents are sensitive to cost; a JPY 1,000 increase in the proposed payment is associated with roughly a 20% reduction in the odds of acceptance (odds ratio ≈ 0.80 per JPY 1,000). Among self‐reported heat experiences during the previous summer, reduced sleep quality shows the largest positive coefficient among the binary experience indicators (*β* = 0.379, OR = 1.461, *p* < 0.01), followed by difficulty performing outdoor activities (*β* = 0.246, OR = 1.279, *p* < 0.05). Notably, acute symptoms—including heatstroke‐like symptoms experienced outdoors or indoors, sunburn, and loss of appetite—are not statistically significant in this specification. Burden of increased electricity costs is negatively associated with acceptance (*β* = −0.319, OR = 0.727, *p* < 0.01), indicating that private cooling expenditure is negatively associated with support for public adaptation investment, consistent with a crowding‐out mechanism.

Risk perception and knowledge variables are also systematically associated with acceptance. The perception that unprecedented extreme heat is likely to occur is positive and statistically significant (*β* = 0.277, OR = 1.319, *p* < 0.01), and greater familiarity with the Heatstroke Alert system, now entered as the 1–4 ordinal scale, is positively associated with acceptance (*β* = 0.337, OR = 1.400, *p* < 0.01). Familiarity with the wet bulb globe temperature (WBGT) shows a non‐monotonic association: Relative to respondents with intermediate familiarity, both those unaware of WBGT (*β* = −0.285, OR = 0.752, *p* < 0.05) and those highly familiar with it (*β* = −0.526, OR = 0.591, *p* < 0.05) are less likely to accept the payment. Demographic variables show systematic differences: young adults (18–34) are significantly less likely to accept the levy (*β* = −0.302, OR = 0.739, *p* < 0.05), whereas elderly respondents (65+) are substantially more likely to accept (*β* = 0.595, OR = 1.813, *p* < 0.01). Residing in a single‐family home is positively associated with acceptance (*β* = 0.280, OR = 1.323, *p* < 0.05), and risk tolerance is also positive (*β* = 0.092, OR = 1.097, *p* < 0.01). Education is negatively associated with acceptance (*β* = −0.117, OR = 0.890, *p* < 0.05). Household income enters as indicators for low (below JPY 4 million), high (JPY 8 million or more), and unreported income, with middle income as the reference category; income level is not jointly significant (Wald *χ* ^2^(2) = 1.1, *p* = 0.58), indicating no detectable independent association between income level and acceptance, whereas respondents who did not report income are less likely to accept (*β* = −0.352, OR = 0.703, *p* < 0.05). Gender is not statistically significant in this model.

When BWS‐derived preference parameters are entered as covariates in the acceptance model, a striking and theoretically informative pattern emerges. The preference parameter for *Strengthening emergency medical services*—the most prioritized countermeasure by a wide margin in both BWS counts and ML estimation—is not statistically significant in the CV model (*β* = −0.004). Similarly, the preference parameters for *Expanding public cooling shelters* (*β* = 0.018) and disseminating real‐time heat information (*β* = −0.005) show non‐significant coefficients. In other words, respondents who more strongly prioritize these three highest‐ranked measures show no statistically detectable difference in their likelihood of accepting the proposed tax increase. Formal Wald tests support this asymmetry: The three most‐prioritized measures are jointly indistinguishable from zero (*χ* ^2^(3) = 0.6, *p* = 0.90), whereas the three lower‐ranked supplementary measures are jointly significant (*χ* ^2^(3) = 17.9, *p* < 0.001), and a linear contrast shows that the mean coefficient of the three lower‐ranked measures differs significantly from that of the three highest‐ranked measures (*χ* ^2^(1) = 7.0, *p* = 0.008). Conversely, preference parameters for lower‐ranked, supplementary measures show positive effects on acceptance, of which public education and water‐refill stations are individually significant while urban greening is positive but does not reach the 5% level: *Implementing public education on heat prevention* (*β* = 0.115, OR = 1.121, *p* < 0.01), *Installing public water refill stations* (*β* = 0.064, OR = 1.066, *p* < 0.05), and *Promoting urban greening* (*β* = 0.040, OR = 1.041). This systematic inversion—whereby lower‐priority measures are positively associated with acceptance while the most‐valued measures show no statistically detectable association—is consistent with what we term the “priority‐WTP disconnect” that the hybrid BWS–CV framework is uniquely positioned to detect.

**FIGURE 5:**
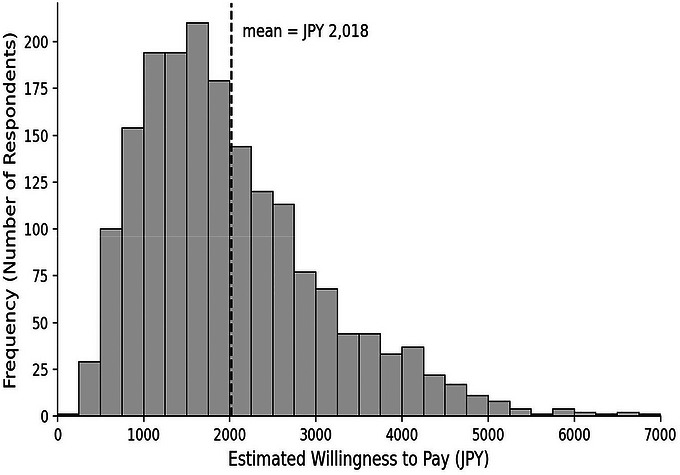
Distribution of estimated willingness to pay (WTP) for public heat countermeasures.

Figure 5 presents the distribution of implied WTP for the proposed policy package, computed from the estimated acceptance model using Equation (5). Among 1,819 respondents, mean implied WTP is JPY 2,018 (SD = 1,112; approximately USD 12.8) with a median of JPY 1,752, ranging from JPY 281 to JPY 8,395. The distribution is right‐skewed, with the mean exceeding the median. The interquartile range spans from JPY 1,180 (25th percentile) to JPY 2,578 (75th percentile), indicating that the middle half of respondents is concentrated within a relatively narrow band, while the 10th percentile (JPY 833) and 90th percentile (JPY 3,502) characterize the distribution tails. This moderate aggregate WTP, combined with the finding that the priority parameter for the most valued measure shows no statistically detectable association with payment acceptance, suggests that the fiscal support available through the proposed tax mechanism may be insufficient for comprehensive adaptation without complementary funding mechanisms.

## 4 Discussion

### 4.1 Immediate Protection Over Long‐Term Prevention

The empirical results demonstrate a decisive survival‐first pattern in risk prioritization. Citizens overwhelmingly prioritize emergency medical services and cooling shelters—measures offering immediate physiological protection—over structural modifications such as urban greening and shading that accumulate benefits over decades. This finding extends the psychometric paradigm (Slovic 1987) to the domain of climate adaptation portfolio choice: When forced to allocate scarce resources among competing protective options, the public treats extreme heat as an imminent survival threat demanding immediate response capacity rather than an environmental condition amenable to gradual structural change. The strength and consistency of this preference—emergency medical services receives a mean coefficient more than twice that of any structural measure—suggests that the survival‐first hierarchy is not a marginal tendency but a dominant cognitive orientation in how citizens evaluate heat risk.

The temporal mismatch between countermeasure types provides the simplest explanation for this pattern. Emergency medical services and cooling shelters deliver certain and immediate risk reduction: A person experiencing heat distress receives treatment today, and a cooling shelter provides measurable physiological relief within minutes of entry. Urban greening, by contrast, requires sustained investment over years before yielding perceptible ambient cooling, and the attribution of any observed temperature reduction to specific greening interventions is inherently uncertain (Stewart and Oke 2012). In an aging population where physiological vulnerability to heat events is high (Ebi et al. 2021), the deterministic benefit of immediate care is heavily favored over the uncertain, delayed benefit of environmental modification. This pattern is consistent with temporal discounting observed in natural hazard preparedness more broadly, where certain near‐term protection is systematically preferred over uncertain long‐term risk reduction (Healy and Malhotra 2009). Behavioral economics analyses of regulatory valuation similarly show that individuals weight near‐term, certain outcomes more heavily than distant, uncertain ones, implying that conventional constant‐rate discounting may understate how steeply citizens themselves discount delayed adaptation benefits (Robinson and Hammitt 2011).

Importantly, the ML results reveal that the survival‐first consensus does not extend uniformly across all measures. *Strengthening emergency medical services* commands broad agreement, combining the highest mean coefficient (*β* = 2.66) with a relatively moderate SD (*σ* = 1.93) and thus the lowest coefficient of variation (*σ*/*β* = 0.7). In contrast, *Promoting urban greening* exhibits comparatively high preference heterogeneity, with a moderate mean (*β* = 1.19) but the largest SD (*σ* = 3.10) and the highest coefficient of variation (*σ*/*β* = 2.6), indicating deeply divided support that likely reflects divergent assessments of maintenance burdens, implementation timeframes, and local applicability (Conway et al. 2011). From a risk governance standpoint, the coefficient of variation serves as a descriptive indicator of the relative dispersion of support for each countermeasure: Measures with high dispersion require substantially more deliberative engagement to build consensus, regardless of their mean popularity.

The low ranking of refined alert systems—despite Japan's well‐established Heatstroke Alert infrastructure—speaks to a broader challenge in risk communication. Alert systems ranked last among the eight measures, selected as “worst” approximately three times more frequently than as “best.” This pattern likely reflects warning fatigue, whereby repetitive issuance of generic, broad‐scale alerts desensitizes the public and reduces perceived utility (Lazo et al. 2009; Sutton et al. 2014; Bean et al. 2016). Conversely, the significantly higher preference for real‐time information dissemination suggests a latent demand for actionable, localized intelligence that supports individual decision‐making rather than additional top‐down warnings. This survival‐first preference structure ultimately exposes a tension between expert risk assessments favoring long‐term structural mitigation and societal prioritization of immediate protective capacity. This divergence need not be interpreted as public irrationality; rather, it reflects a rational response to the temporal structure of heat risk as experienced by individuals.

### 4.2 The Preference–WTP Disconnect: Entitlement and Experiential Processing

The most striking finding of this study is the systematic disconnect between risk prioritization and fiscal support. Emergency medical services—ranked first by a wide margin in both BWS counts and ML estimation—shows no statistically detectable association with acceptance in the CV model. Expanding cooling shelters and real‐time information, which rank second and third respectively, also show non‐significant coefficients. The formal joint tests and coefficient contrast indicate that this pattern is not merely a comparison between individually significant and non‐significant coefficients. It is consistent with a systematic asymmetry in how different types of public risk reduction relate to fiscal acceptance, with implications for adaptation financing.

We interpret this pattern through the lens of fiscal entitlement perceptions. Citizens appear to regard life‐saving emergency response as a non‐negotiable government obligation that should be funded through existing general revenue, not through additional household taxation. This interpretation aligns with established research in fiscal psychology showing that public resistance to new taxation increases sharply when the targeted service is perceived as a basic right or essential government function rather than an optional amenity or enhancement (Kirchler 2007; Slemrod 2007). In this cognitive framework, expressing strong priority for emergency medical services signals that the respondent considers it fundamentally important—but precisely because it is so fundamental, the respondent may reject the proposition that personal payment should be required to secure it. The lack of marginal WTP is thus consistent with an entitlement interpretation rather than indifference: A reluctance to accept the payment vehicle for what is perceived as a baseline obligation of governance.

By comparison, the preference parameters for several lower‐ranked supplementary measures are positively associated with payment acceptance. The preference parameters for public education on heat prevention and water refill stations—both ranked lower in the BWS hierarchy—show statistically significant positive associations with acceptance of the tax increase. The coefficient for urban greening is also positive but does not reach the 5% significance level. Collectively, however, these three lower‐ranked measures are jointly associated with acceptance. One possible interpretation is that citizens categorize these measures as “value‐added amenities” that enhance protection beyond the minimum entitlement, making them acceptable candidates for additional discretionary funding. If so, this differential categorization would create an inversion: The measures most valued in terms of priority may be less amenable to new dedicated taxation, while less‐valued supplementary measures are more readily financeable through earmarked levies.

This finding has important methodological implications for risk valuation research. Standard CV studies estimating WTP for a single predefined policy cannot detect entitlement effects because they do not observe the relative priority of the valued item against alternatives. By integrating BWS‐derived priority parameters into the CV acceptance model, the hybrid framework reveals that preference intensity and WTP operate through partially independent cognitive pathways. Relying solely on WTP to infer public risk priorities can therefore be systematically misleading: The most valued protections may generate the lowest marginal WTP precisely because they are perceived as entitlements.

The experiential drivers of fiscal support reinforce the theoretical importance of chronic quality‐of‐life burdens in risk valuation. Reduced sleep quality emerged as the single largest positive coefficient among the binary experience indicators (OR = 1.461), whereas none of the acute‐symptom indicators showed a statistically significant association. This finding is consistent with dual‐process models of risk cognition (Slovic et al. 2004; Weber 2006): Chronic, personally experienced disruption functions as an affective signal that converts abstract climate risk into concrete economic demand for collective action. Physiological research linking elevated nighttime temperatures to measurable sleep degradation (Obradovich et al. 2017) positions sleep disruption as a mediating pathway through which climate change translates into fiscal support through embodied experience rather than statistical knowledge.

The negative association between electricity cost burden and WTP (OR = 0.727, *p* < 0.01) is consistent with a crowding‐out mechanism with potential distributional consequences. Households facing higher private cooling expenditures are significantly less willing to support additional public spending, raising a potential distributional concern, in that households most physically exposed to heat stress—those in poorly insulated housing with high cooling costs (Harlan et al. 2006)—may be among the least likely to support the collective measures from which they would benefit most. Energy affordability may therefore constrain the political feasibility of publicly financed heat adaptation (Liddell and Morris 2010; Bouzarovski and Petrova 2015).

### 4.3 Demographic Patterns and Risk Communication Implications

The age gradient in fiscal support reveals important differences in how heat risk is experienced across demographic groups. Elderly respondents (65+) show substantially higher acceptance of the proposed tax (OR = 1.813, *p* < 0.01), while young adults (18–34) are significantly less supportive (OR = 0.739, *p* < 0.05). This pattern likely reflects differential vulnerability salience: Older adults experience heat stress more acutely due to physiological sensitivity (Ebi et al. 2021; Ng et al. 2016) and are more likely to have witnessed heat‐related emergencies in their communities, generating experiential processing of personal vulnerability consistent with the dual‐process framework described above.

For younger cohorts, heat exposure manifests primarily through productivity and lifestyle constraints rather than direct physiological danger. The finding that sleep disruption and outdoor activity limitations are the strongest experiential drivers of WTP suggests a potential avenue for bridging this age gap: Risk communication that frames heat adaptation as protection of daily functioning and quality of life may resonate more effectively with working‐age populations than messaging centered on mortality reduction (Spence and Pidgeon 2010). The positive association between risk tolerance and acceptance (OR = 1.097, *p* < 0.01) may reflect the nature of the payment mechanism—accepting a new annual tax constitutes a financial risk in itself—and risk‐tolerant individuals may be more comfortable with this uncertainty. The positive effect of single‐family home residence (OR = 1.323, *p* < 0.05) likely reflects higher stakes in neighborhood livability and greater attachment to place.

The role of thermal literacy also merits attention. High awareness of the Heatstroke Alert system is positively associated with acceptance, while for WBGT the relationship is non‐monotonic: Both respondents unaware of it—likely the least engaged with heat risk—and those most familiar with it—likely the most heat‐literate and the most able to manage exposure privately—are less supportive of collective provision than respondents with intermediate familiarity.

### 4.4 Policy Implications

The findings identify four operational principles for designing fiscally viable heat adaptation portfolios, each grounded directly in the empirical patterns observed. Public preferences should not mechanically determine adaptation portfolios or override evidence on technical effectiveness; rather, the observed preference structure identifies where risk communication and deliberative engagement are needed to build support for technically effective measures whose benefits may be delayed or less immediately visible. First, the survival‐first preference hierarchy indicates that immediate protective capacity—emergency response and cooling shelters—should be treated as a politically salient component of adaptation portfolios, while its scale and design should remain guided by technical effectiveness and distributive considerations. However, because these measures are perceived as government obligations, their expansion may be less likely to succeed through new dedicated levies and should instead be financed through general revenue reallocation. Policymakers who attempt to fund essential emergency services through earmarked taxes may face public rejection not because citizens undervalue the service, but because they categorize it as an obligation that existing governance structures should already fulfill.

Second, measures exhibiting high preference heterogeneity—particularly urban greening, with a coefficient of variation of 2.6—require substantially more deliberative public engagement before implementation than measures with broader consensus. The dispersion of preferences, not merely the mean, should inform the sequencing and communication of adaptation investments. A measure with moderate average support but extreme polarization may require targeted consultation, pilot programs, or design modifications before achieving sufficient political viability for large‐scale rollout. The SD/mean ratio thus provides a practical diagnostic tool for adaptation planning.

Third, the negative association between electricity‐cost burden and acceptance suggests that uniform adaptation charges may raise distributional concerns. Complementary measures addressing energy affordability—such as efficiency subsidies, insulation programs, or differentiated pricing structures—may be necessary to maintain fiscal support that is widely shared across income groups for collective heat protection (McCauley and Heffron 2018). More broadly, adaptation portfolios should be designed with explicit attention to how public funding mechanisms interact with existing private expenditure patterns. A policy package that ignores energy affordability may succeed technically while failing politically. If private cooling burdens are concentrated among vulnerable households, those most in need of collective protection may also face greater difficulty bearing additional costs.

Fourth, the distinction between entitlement‐category and amenity‐category measures has implications for how adaptation packages are communicated and financed. Risk communication that frames all heat countermeasures as discretionary public goods may generate backlash if the most valued measures are perceived as existing governmental obligations. Conversely, transparently separating “baseline protections” financed through general revenue from “enhanced services” supported by dedicated fees may improve both fiscal clarity and public acceptance. This dual‐track financing approach aligns funding mechanisms with the cognitive categories that citizens actually use when evaluating risk reduction, rather than imposing a uniform framework that contradicts those categories.

### 4.5 Limitations and Future Research

Several limitations of this study warrant acknowledgment and suggest directions for future investigation. First, the survey was conducted in March, which successfully avoids the “focusing illusion” that might inflate risk salience during peak summer conditions, but introduces potential recall bias regarding the severity of specific symptoms experienced during the previous summer. The survival‐first preference might be even more pronounced if measured during an active heat event above 35°C, or it might shift if measured during winter when heat is a distant concern. Future research should employ longitudinal tracking across seasons to capture the dynamic temporal elasticity of public risk prioritization.

Second, the use of an online panel, while enabling the interactive choice tasks essential for BWS implementation, resulted in a sample with higher educational attainment compared to the census population. This composition likely inflates absolute WTP estimates. More importantly, the online format inherently excludes digitally disconnected populations—who are disproportionately elderly, low‐income, and vulnerable to both heat exposure and energy poverty. Supplementing online methods with in‐person interviews in future studies would strengthen external validity and ensure representation of the most marginalized voices.

Third, the key experiential variable—reduced sleep quality—relied on self‐reporting, which introduces measurement uncertainty regarding both the severity and the causal attribution of sleep disruption. Integrating stated preference surveys with objective physiological monitoring through wearable sleep trackers would allow more precise calibration of how physical heat stress translates into economic valuation, potentially revealing nonlinear thresholds at which sleep degradation triggers fiscal willingness.

Fourth, while the single‐city design provides detailed contextual analysis of a compelling case, generalizability across different climatic zones, institutional contexts, and cultural settings requires comparative investigation. The survival‐first hierarchy observed in Yokohama may reflect the specific combination of advanced aging, dense urbanization, and humid subtropical climate that characterizes Japanese megacities. Whether the same preference structure holds in cities with different demographic profiles, climate types, or governance traditions remains an open empirical question.

Finally, the entitlement interpretation of the preference–WTP disconnect, while theoretically grounded in fiscal psychology and consistent with the observed patterns, would benefit from direct qualitative investigation. Focus groups or deliberative exercises that probe the reasoning behind citizens’ simultaneous prioritization of emergency services and reluctance to accept additional taxation for them could clarify the underlying cognitive mechanisms and inform the design of financing strategies that do not conflict with public perceptions of governmental obligation.

Two methodological caveats also warrant mention. The individual BWS priority parameters are posterior estimates rather than directly observed covariates. Because the second‐stage logistic regression treats these generated regressors as fixed, it may understate the uncertainty associated with the estimated priorities. In addition, because implied WTP is derived from a linear‐in‐bid acceptance model, individual values above the maximum offered bid of JPY 3000 rely on extrapolation beyond the observed bid range and should be interpreted with particular caution.

A further caveat concerns possible restriction of range. The absence of a statistically detectable association between the highest‐prioritized measure—emergency medical services—and acceptance may partly reflect the unusually broad consensus on its importance, which leaves limited cross‐respondent variation for the acceptance model to exploit. This explanation cannot fully account for the observed pattern, however, because the other two highly ranked measures—public cooling shelters and real‐time heat information—exhibit substantial preference heterogeneity yet likewise show no significant association with acceptance. The entitlement interpretation therefore remains a theoretically consistent explanation for the broader joint pattern.

## 5 Conclusions

Using a hybrid BWS and CV framework applied to 1,819 residents of Yokohama, Japan, this study provides three principal findings relevant to public risk prioritization under climate change.

First, citizens exhibit a clear survival‐first hierarchy when asked to prioritize competing heat countermeasures under fiscal constraints. Immediate protective measures—emergency medical services and cooling shelters—receive decisively higher priority than long‐term structural interventions such as urban greening. Importantly, consensus varies markedly across measures: Emergency medical services commands broad agreement (low coefficient of variation), while urban greening shows comparatively high preference heterogeneity (highest coefficient of variation). This hierarchy reveals that the public frames urban heat primarily as a survival risk demanding immediate response rather than an environmental condition amenable to gradual structural modification.

Second, a systematic disconnect exists between risk prioritization and fiscal support. The most highly prioritized measure—emergency medical services—showed no statistically detectable association with WTP in the CV model, consistent with an entitlement perception in which life‐saving services are viewed as a non‐negotiable government obligation rather than a service warranting additional personal taxation. Conversely, a group of lower‐ranked supplementary measures—public education, water refill stations, and urban greening—are jointly associated with greater fiscal support, although only the public‐education and water‐refill coefficients are individually significant at the 5% level. This pattern suggests that preference intensity and WTP may operate through partially distinct evaluative pathways, and that standard valuation studies relying on WTP alone may systematically misrepresent the structure of public risk priorities.

Third, fiscal support for collective heat adaptation was more consistently associated with chronic quality‐of‐life burdens—particularly sleep disruption—than with the measured acute symptoms. Reduced sleep quality showed the largest positive coefficient among the experiential indicators, whereas none of the acute‐symptom indicators was statistically significant. Concurrently, electricity‐cost burden was negatively associated with acceptance, consistent with a possible crowding‐out mechanism; if such burdens are concentrated among lower‐income or poorly insulated households, this relationship may create distributional barriers to equitable adaptation finance. This interaction between private coping costs and public willingness to invest represents a structural barrier to equitable climate adaptation that financing strategies must explicitly address.

These findings collectively reframe heat adaptation from a purely technical allocation problem to a risk governance challenge involving prioritization, entitlement perceptions, and distributional equity. Designing adaptation portfolios that are both effective and socially sustainable requires understanding and aligning with how citizens cognitively categorize different types of risk reduction—distinguishing between entitlements that demand general revenue and amenities that can sustain dedicated financing. The hybrid BWS–CV methodology provides a practical tool for detecting these priority–fiscal support patterns and informing adaptation governance accordingly.

## Supporting information




**Supporting Information Table S1**. Variance inflation factors for the Table 6 covariates.
**Supporting Information Table S2a**. Correlations among heat‐experience items.
**Supporting Information Table S2b**. Correlations among BWS preference parameters.
**Supporting Information Table S3a**. Factor loadings and uniqueness — heat‐experience items (tetrachoric, principal factors).
**Supporting Information Table S3b**. Factor loadings and uniqueness — preference parameters (principal‐component factors).
**Supporting Information Figure S1**. Scree plot, heat‐experience items.
**Supporting Information Figure S2**. Scree plot, preference parameters.
**Supporting Information Table S4**. Household‐income indicators in the acceptance model (Table 6 specification).

## Endnote

1 JPY 1,000 corresponds to approximately USD 6.17 and EUR 5.41 (As of June 30, 2026).
