# Supplementary material for: Survival First: How Citizens Prioritize Competing Climate‐Health Risk Countermeasures Under Fiscal Constraints
Source: Risk Anal. 2026 Jul 24;46(8):e70315. doi: 10.1111/risa.70315 (PMC13396970; doi:10.1111/risa.70315)
Supplement: Supplementary file 1 — Supporting Information Table S1. Variance inflation factors for the Table 6 covariates. Supporting Information Table S2a. Correlations among heat‐experience items. Supporting Information Table S2b. Correlations among BWS preference parameters. Supporting Information Table S3a. Factor loadings and uniqueness — heat‐experience items (tetrachoric, principal factors). Supporting Information Table S3b. Factor loadings and uniqueness — preference parameters (principal‐component factors). Supporting Information Figure S1. Scree plot, heat‐experience items. Supporting Information Figure S2. Scree plot, preference parameters. Supporting Information Table S4. Household‐income indicators in the acceptance model (Table 6 specification). [file RISA-46-0-s001.docx]

**Supplementary Materials**

*Manuscript: “Survival first: How citizens prioritize competing climate-health risk countermeasures under fiscal constraints.”*

These diagnostics accompany the acceptance model reported in Table 6. They show that the model’s predictors are not multicollinear and that the heat-experience items and the countermeasure-preference parameters cannot be reduced to theoretically coherent and empirically well-defined composite scales suitable for the acceptance model; we therefore retain the individual variables.

**S1. Multicollinearity (variance inflation factors)**

Variance inflation factors (VIF) were computed for the full set of Table 6 covariates. The maximum VIF is 1.45 and the mean is about 1.1; all values are far below conventional thresholds of concern (5 or 10), indicating no evidence of problematic multicollinearity.

**Table S1. Variance inflation factors for the Table 6 covariates.**

| **Variable** | **VIF** |  | **Variable** | **VIF** |
| --- | --- | --- | --- | --- |
| Annual payment | 1.02 |  | Pref: Public education | 1.04 |
| Outdoor-activity difficulty | 1.08 |  | Perception: extreme heat likely | 1.08 |
| Work performance decline | 1.13 |  | Heatstroke-Alert familiarity (1-4) | 1.36 |
| Heatstroke sympt. (outdoor) | 1.19 |  | Unaware of WBGT | 1.20 |
| Heatstroke sympt. (indoor) | 1.12 |  | Highly familiar with WBGT | 1.16 |
| Sunburn/skin | 1.11 |  | Male | 1.15 |
| Reduced sleep quality | 1.10 |  | Young (18-34) | 1.14 |
| Appetite/weight change | 1.09 |  | Elderly (65+) | 1.23 |
| Electricity-cost burden | 1.03 |  | Single-family home | 1.06 |
| Pref: Emergency medical | 1.03 |  | Risk tolerance | 1.08 |
| Pref: Cooling shelters | 1.12 |  | Income: low (<4M) | 1.45 |
| Pref: Real-time info | 1.12 |  | Income: high (>=8M) | 1.30 |
| Pref: Urban greening | 1.09 |  | Income: not reported | 1.38 |
| Pref: Shaded areas | 1.10 |  | Education | 1.04 |
| Pref: Water refill | 1.09 |  |  |  |
|  |  |  |  |  |

**S2. Pairwise correlations**

The largest absolute pairwise correlation is 0.27 among the heat-experience items (Table S2a) and 0.22 among the BWS preference parameters (Table S2b). These modest correlations are consistent with the low VIFs in Table S1.

**Table S2a. Correlations among heat-experience items.**

|  | **1** | **2** | **3** | **4** | **5** | **6** | **7** | **8** |
| --- | --- | --- | --- | --- | --- | --- | --- | --- |
| 1. Outdoor difficulty | 1.00 |  |  |  |  |  |  |  |
| 2. Work decline | 0.12 | 1.00 |  |  |  |  |  |  |
| 3. Heatstroke outdoor | 0.16 | 0.15 | 1.00 |  |  |  |  |  |
| 4. Heatstroke indoor | 0.07 | 0.15 | 0.27 | 1.00 |  |  |  |  |
| 5. Sunburn/skin | 0.12 | 0.12 | 0.17 | 0.08 | 1.00 |  |  |  |
| 6. Sleep quality | 0.06 | 0.17 | 0.13 | 0.11 | 0.12 | 1.00 |  |  |
| 7. Appetite/weight | 0.06 | 0.15 | 0.17 | 0.13 | 0.11 | 0.16 | 1.00 |  |
| 8. Electricity cost | -0.03 | 0.03 | 0.02 | 0.01 | 0.06 | 0.06 | 0.02 | 1.00 |
|  |  |  |  |  |  |  |  |  |

**Table S2b. Correlations among BWS preference parameters.**

|  | **1** | **2** | **3** | **4** | **5** | **6** | **7** |
| --- | --- | --- | --- | --- | --- | --- | --- |
| 1. Greening | 1.00 |  |  |  |  |  |  |
| 2. Shade | 0.05 | 1.00 |  |  |  |  |  |
| 3. Water | -0.15 | 0.10 | 1.00 |  |  |  |  |
| 4. Cooling | -0.11 | 0.19 | 0.14 | 1.00 |  |  |  |
| 5. Real-time info | -0.11 | -0.22 | -0.10 | -0.19 | 1.00 |  |  |
| 6. Education | 0.03 | -0.08 | -0.08 | -0.06 | 0.08 | 1.00 |  |
| 7. Emergency | -0.02 | -0.06 | -0.04 | 0.01 | -0.01 | 0.08 | 1.00 |
|  |  |  |  |  |  |  |  |

**S3. Factor analyses**

We conducted exploratory factor analyses of (i) the eight heat-experience items (tetrachoric correlations, principal-factor extraction) and (ii) the seven countermeasure-preference parameters (principal-component factors).

**Heat-experience items.** The scree plot and eigenvalues indicate a single weak common factor (Factor 1 eigenvalue ≈ 1.9; the next eigenvalue ≈ 0.2); for diagnostic transparency we display the first three unrotated factors in Table S3a. The loadings are weak and uniquenesses are high (0.49–0.94; the electricity-cost item barely loads), so most variance is item-specific. Substantively, items loading on the same factor have opposite-signed associations with acceptance in Table 6 (reduced sleep quality positive; electricity-cost burden negative), so a single scale would cancel these opposing mechanisms.

**Preference parameters.** Three factors have eigenvalues above one (1.53, 1.17, 1.06), indicating that the preferences are genuinely multidimensional and cannot be represented by a single index.

**Table S3a. Factor loadings and uniqueness — heat-experience items (tetrachoric, principal factors).**

| **Experience item** | **Factor 1** | **Factor 2** | **Factor 3** | **Uniqueness** |
| --- | --- | --- | --- | --- |
| Outdoor-activity difficulty | 0.354 | -0.221 | 0.193 | 0.788 |
| Work performance decline | 0.502 | 0.085 | 0.009 | 0.741 |
| Heatstroke symptoms (outdoor) | 0.688 | -0.184 | -0.042 | 0.491 |
| Heatstroke symptoms (indoor) | 0.591 | -0.112 | -0.197 | 0.600 |
| Sunburn / skin problems | 0.474 | 0.042 | 0.209 | 0.730 |
| Reduced sleep quality | 0.452 | 0.243 | 0.000 | 0.737 |
| Appetite / weight change | 0.542 | 0.147 | -0.059 | 0.682 |
| Electricity-cost burden | 0.090 | 0.211 | 0.057 | 0.944 |
|  |  |  |  |  |

**Table S3b. Factor loadings and uniqueness — preference parameters (principal-component factors).**

| **Preference parameter** | **Factor 1** | **Factor 2** | **Factor 3** | **Uniqueness** |
| --- | --- | --- | --- | --- |
| Urban greening | -0.105 | 0.833 | -0.056 | 0.291 |
| Shaded areas | 0.620 | 0.287 | 0.006 | 0.533 |
| Water refill | 0.488 | -0.445 | -0.041 | 0.562 |
| Cooling shelters | 0.629 | -0.168 | 0.279 | 0.499 |
| Real-time information | -0.603 | -0.407 | -0.195 | 0.433 |
| Public education | -0.351 | 0.050 | 0.523 | 0.601 |
| Emergency medical | -0.121 | -0.039 | 0.814 | 0.321 |
|  |  |  |  |  |


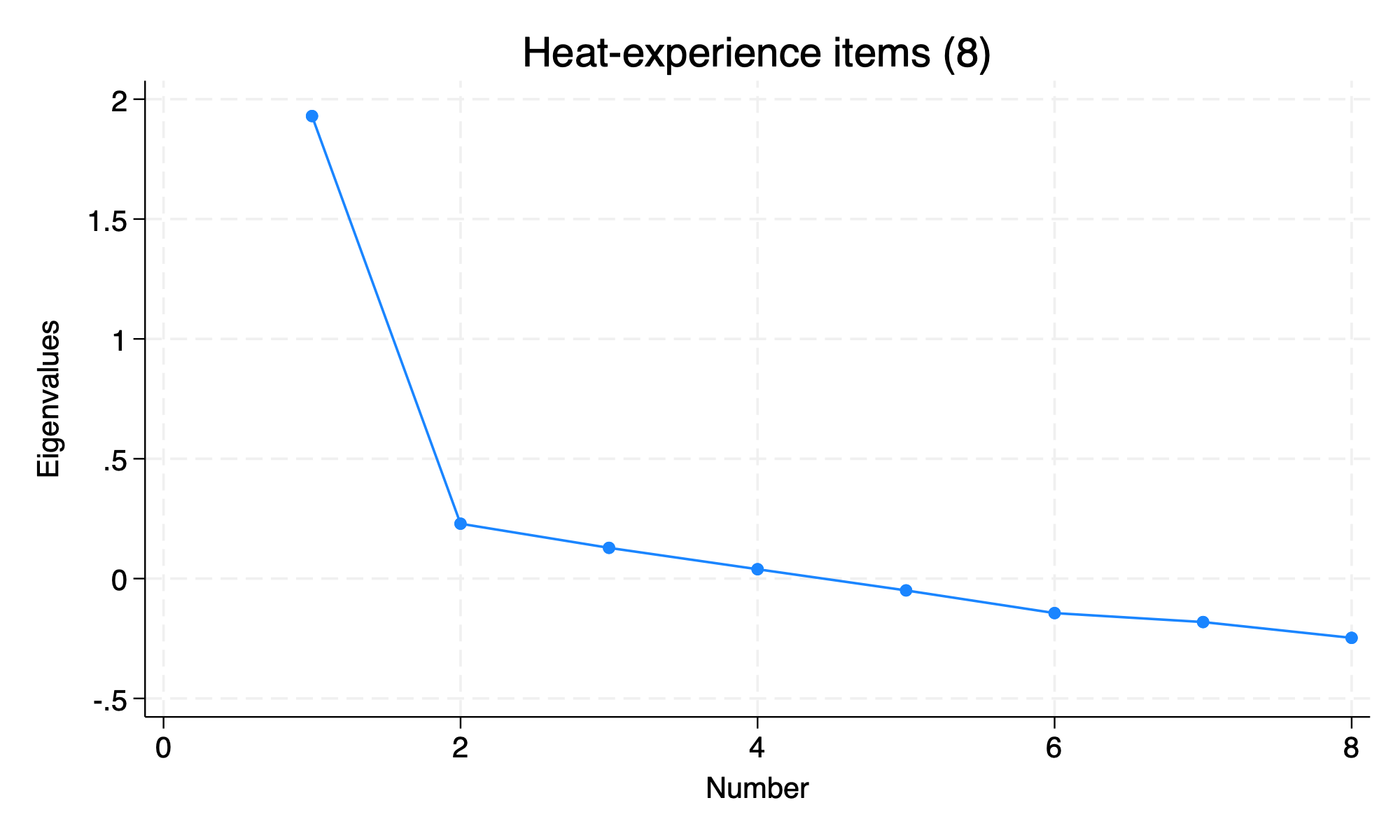


**Figure S1. Scree plot, heat-experience items.**


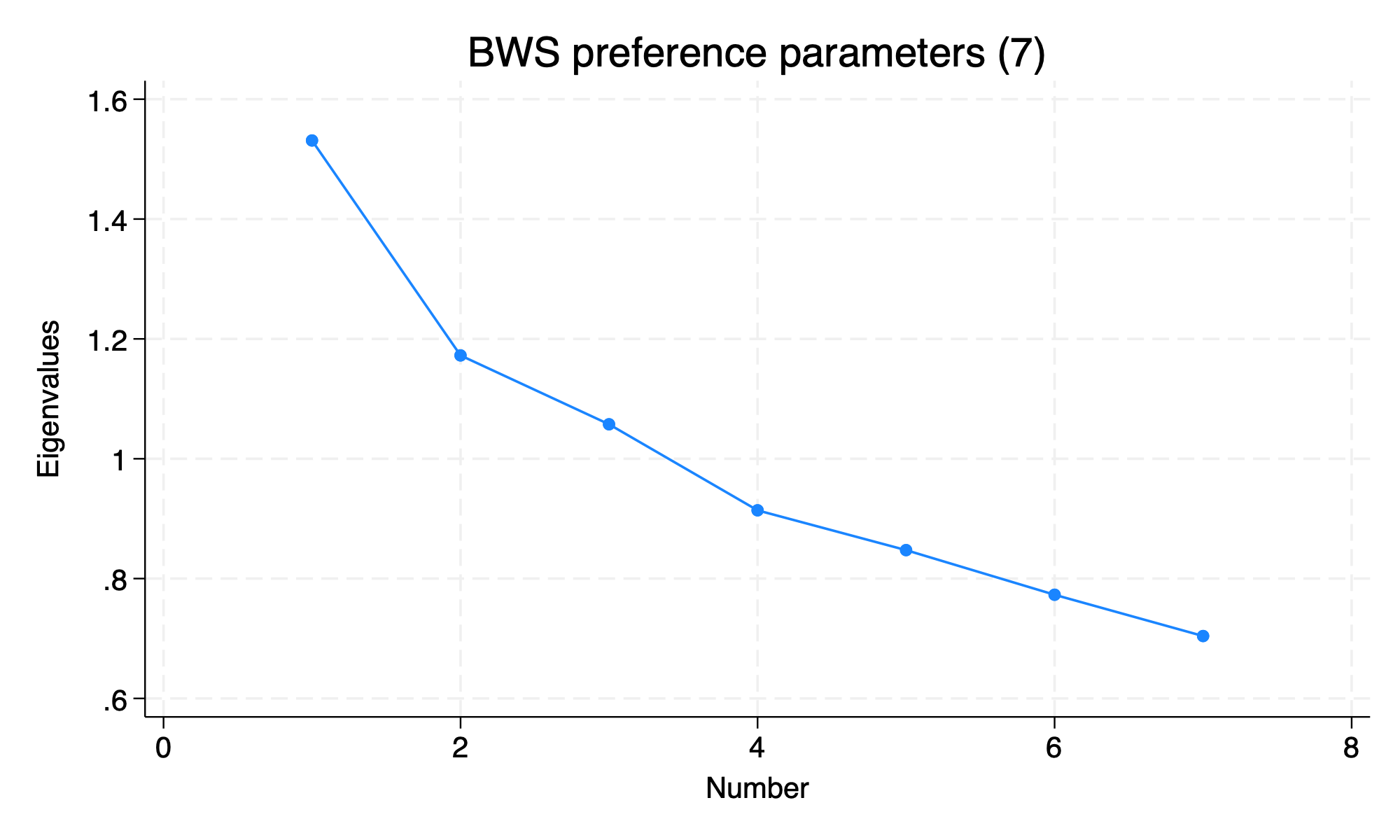


**Figure S2. Scree plot, preference parameters.**

**S4. Household income (Reviewer 1)**

Reviewer 1 asked that household income be considered. Because 22.2% of respondents declined to report income, we did not impute a continuous measure; instead income enters as three indicators—low (below JPY 4 million), high (JPY 8 million or more), and not reported—with middle income (JPY 4–8 million) as the reference category, so that all 1,819 observations are retained without imputation. This specification separates the effect of income level from that of non-response. As shown in Table S4, income level is not jointly significant (Wald χ²(2) = 1.08, p = 0.58): neither lower- nor higher-income households differ significantly from middle-income households, so we find no statistically detectable independent association between income category and acceptance. The only significant income-related term is non-response (OR = 0.70, p < 0.05): respondents unwilling to disclose income are less likely to accept the tax, which may reflect broader fiscal or privacy reticence, although this cannot be tested directly with the present data.

**Table S4. Household-income indicators in the acceptance model (Table 6 specification).**

| **Income indicator (reference = middle income, JPY 4–8 million)** | **Number coded 1** | **Odds ratio** | **p-value** |
| --- | --- | --- | --- |
| Low income (below JPY 4 million) | 530 | 0.906 | 0.460 |
| High income (JPY 8 million or more) | 294 | 1.069 | 0.671 |
| Income not reported | 403 | 0.703 | 0.012 |
|  |  |  |  |

Note: Estimated within the full Table 6 model. The reference category is middle income (JPY 4–8 million). The joint test that the low- and high-income coefficients are both zero gives Wald χ²(2) = 1.08 (p = 0.58).
